# An Improved DBSCAN Method for LiDAR Data Segmentation with Automatic Eps Estimation

**DOI:** 10.3390/s19010172

**Published:** 2019-01-05

**Authors:** Chunxiao Wang, Min Ji, Jian Wang, Wei Wen, Ting Li, Yong Sun

**Affiliations:** 1 Geomatics College, Shandong University of Science and Technology, Qingdao 266590, China; cx8989@163.com (C.W.); rainbowwj@126.com (J.W.); liting_sdust@126.com (T.L.); ttsunyong@163.com (Y.S.); 2 Hainan Geomatics Centre, National Administration of Surveying, Mapping and Geoinformation of China, Haikou 570203, China; wenwei19821010@126.com

**Keywords:** LiDAR, segmentation, DBSCAN, parameter estimation

## Abstract

Point cloud data segmentation, filtering, classification, and feature extraction are the main focus of point cloud data processing. DBSCAN (density-based spatial clustering of applications with noise) is capable of detecting arbitrary shapes of clusters in spaces of any dimension, and this method is very suitable for LiDAR (Light Detection and Ranging) data segmentation. The DBSCAN method needs at least two parameters: The minimum number of points *minPts*, and the searching radius ε. However, the parameter ε is often harder to determine, which hinders the application of the DBSCAN method in point cloud segmentation. Therefore, a segmentation algorithm based on DBSCAN is proposed with a novel automatic parameter ε estimation method—Estimation Method based on the average of k nearest neighbors’ maximum distance—with which parameter ε can be calculated on the intrinsic properties of the point cloud data. The method is based on the fitting curve of k and the mean maximum distance. The method was evaluated on different types of point cloud data: Airborne, and mobile point cloud data with and without color information. The results show that the accuracy values using ε estimated by the proposed method are 75%, 74%, and 71%, which are higher than those using parameters that are smaller or greater than the estimated one. The results demonstrate that the proposed algorithm can segment different types of LiDAR point clouds with higher accuracy in a robust manner. The algorithm can be applied to airborne and mobile LiDAR point cloud data processing systems, which can reduce manual work and improve the automation of data processing.

## 1. Introduction

LiDAR (Light Detection and Ranging) technology has the advantages of high data density, high precision, high operation efficiency, and strong penetrating power. In addition to traditional field surveying and remote sensing [1], LiDAR technology is widely used in many other areas, such as forest ecology [2,3,4,5], urban change detection [6], urban road detection, and planning [7,8], robot environment perception [9], and autopilot technology [10], in which it has played an increasingly important role. However, interpreting the LiDAR point cloud data remains a fundamental research challenge. Laser scanning technology is a new space for ground observation technology but compared to the rapid development of laser scanning system hardware, point cloud data processing and application of the study are lagging behind. At present, although a series of research results have been presented in the study of point cloud segmentation, filtering, classification, and feature extraction, these methods are mainly applicable to certain datasets or need the user to have a good prior understanding of the data. Fast and automatic high-precision segmentation is still difficult to achieve using the current point cloud data processing methods.

In this study, we focus on automatic point cloud segmentation, and a DBSCAN (density-based spatial clustering of applications with noise) parameter estimation method is proposed. The method is evaluated on different types of point cloud data and it is shown to perform well in airborne and mobile LiDAR data experiments.

## 2. Previous Work on LiDAR Data Segmentation

Point cloud data segmentation is the basis for scene reconstruction and object identification. It is also a key step of point cloud data processing. The current mainstream of the point cloud segmentation algorithm is mainly based on a clustering algorithm, model fitting algorithm, region-growing algorithm, and other segmentation methods.

### 2.1. Clustering-Based Method

Density-based clustering methods, such as DBSCAN [11], OPTICS (ordering points to identify the clustering structure) [12], and DENCLUE (density-based clustering) [13], among others, are capable of detecting arbitrary shaped clusters [14]. LiDAR data clusters have arbitrary shapes and therefore densities, and therefore clustering methods are applicable for segmenting LiDAR data [15].

However, there are few studies showing the efficiency and auto-segmentation of the DBSCAN method for LiDAR data segmentation. The radius parameter ε is often difficult to set. Current studies using DBSCAN for LiDAR segmentation mainly focus on specific data. For example, each LiDAR dataset has to be well understood, and the parameter value ε should be chosen carefully. Given the way DBSCAN is designed, ε should be as small as possible. The value of ε also depends on the distance function. In an ideal scenario, there exists domain knowledge to choose a parameter that is based on the application domain [16]. That is, the user has to understand the data very well and choose ε very carefully. This situation hinders the application of DBSCAN in automatic clustering.

Ester et al. [11] attempted to develop a simple and effective heuristic to determine the parameters ε and *minPts* of the algorithm using k-distance graphs. The k-distance graphs map each point to the distance of the *k*-th nearest neighbor. The authors argue that with two-dimensional data, for all *k* > 4, the graphs do not significantly differ from a 4-distance graph. However, this process is rather interactive, and the authors recommend using the graphical representation of the k-distance graph to help users estimate the correct threshold. In generalized DBSCAN, Sander et al. [17] used the *k*-distance graph to determine the parameters and suggested using the (2·dim−1) nearest neighbor and minPts=2·dim (dim is the dataset dimensionality), while the value of k in the k-distance graph has to be given by the user. Daszykowski, Walczak, and Massart have attempted to establish rules of thumb for chemical applications, however, the rules have to be tested for LiDAR data [18].

Although parameter estimation methods have been studied, there are also some different views on the same estimation methods. For example, for the clustering of a household data set normalized to integers in $\left[0;10^{5}\right]$ from the UCI (University of California, Irvine) machine learning repository [19], Schubert and Sander suggested that the minimum value of ε should clearly be chosen below ε < 2000 [18], while Gan and Tao suggested using ε ≥ 5000 instead [20].

Ghosh and Lohani examined the effect of DBSCAN and OPTICS on LiDAR data. They showed that DBSCAN performed comparatively better as shown by the ARI (Adjusted Rand Index, a measure of the similarity between two different clustering approaches, proposed by Hubert and Arabie [21]). The thresholds used in this study were determined by running an experiment with the thresholds from a low value of 0.7 m to a high value of 4.0 m [15,22]. An analytical study for the automatic determination of the thresholds using the parameters of the LiDAR data is therefore strongly recommended [22]. Lari and Habib took the 3D relationship among the points and the physical properties of the surfaces they belong to into account for adaptive LiDAR data processing [23].

Many segmentation research papers are based on another clustering method. Biosca and Lerma proposed a planar extraction algorithm based on the fuzzy C-means algorithm (FCM) [24]. Filin proposed a surface clustering algorithm that realized house and vegetation point cloud segmentation [25]. Jiang proposed a self-organizing maps (SOM) algorithm and applied it to point cloud feature extraction [26], which can be used for unsupervised classification without prior knowledge, but the learning process of this method is still dependent on the input parameters. Morsdorf et al. used a K-means clustering algorithm to realize the extraction of single trees in the woods in airborne point cloud data [27]. Roggero used a three-dimensional tensor to generate the n-dimensional eigenvector and used the hierarchical clustering algorithm to segment the airborne cloud data [28]. Biosca and Lerma proposed a planar extraction algorithm based on fuzzy clustering with the fuzzy C-means (FCM) [24]. Crosilla et al. used the second-order Taylor expansion to detect Gaussian curvature, the mean curvature from the neighborhood point set, and divided the point cloud into regular geometries by clustering [29]. The commonly used spatial segmentation methods are K Nearest Neighbors (KNN) and the maximum likelihood method. Jain and Duin et al. summarized several other methods of statistical pattern recognition [30].

In general, in the field of laser point cloud data segmentation, scholars have undertaken a lot of research and made a lot of achievements. However, most of these clustering-based segmentation methods apply only to some specific data. Most methods rely on manual experience, while fewer can achieve automatic segmentation. Some clustering methods are very sensitive to input parameters, and small differences can lead to completely different clustering results. Although the above researchers have achieved good experimental results, their segmentation accuracy depends on the artificial definition of the segmentation parameters, which are mostly related to the equipment and the specific data.

Based on these studies, a parameter estimation method based on the DBSCAN density clustering method is proposed and is described in detail in Section 3.2.

### 2.2. Model Fitting-Based Method

The two category approaches of model fitting-based methods are the Hough Transform (HT) [31,32] and the Random Sample Consensus (RANSAC) approach proposed by Fischler and Bolles (1981) [33]. The HT method is used to detect planes, cylinders, and spheres in the point cloud. Hoffman and Jain [34] summarized three basic forms of boundary in laser point cloud data: Jump edges, crease edges, and smooth edges. Based on these basic forms, model fitting-based methods have been developed. Yang et al. proposed a two-step adaptive extraction method for ground points and break lines from LiDAR point clouds [35]. Maas and Vosselman reconstructed the regular building model with the invariant moment method [36].

In the RANSAC method, candidate shape primitives are used to check against all points to determine the best model fit [33]. This method has been used in point cloud segmentation. For example, Riveiro et al. used the automatic detection method based on road surface segmentation to find zebra crossings from mobile LiDAR data [37]. Neidhart used the original LiDAR point cloud data to extract building information relating to elevation and geometry, then reconstructed the building using a graphical approach [38]. Woo et al., Su et al. and Vo et al. proposed point cloud data segmentation methods based on the octree-based three-dimensional lattice to handle a large number of disordered point datasets [39,40,41]. Boulaassal et al. used the RANSAC algorithm to extract building facade planes from terrestrial laser scanner data [42]. Schnabel et al. used RANSAC to test the shape of scattered cloud points by random sampling of planes, spheres, cylinders, and other shapes [43]. Awwad et al. improved the RANSAC algorithm by dividing the dataset into small clusters based on normal vectors of the points [44]. Schwalbe et al. used two or more neighboring planes in groups, and 2D GIS (Geographic Information System) data, to generate a 3D building model [45]. Moosmann et al. used a graph-based approach to segment the ground and objects from 3D LiDAR scans using a unified, generic criterion based on local convexity measures [46]. Segmentation of dense 3D data (e.g., Riegl scans) was optimized via a simple efficient voxelization of the space [47].

The HT and RANSAC methods are robust methods for point cloud segmentation, and the RANSAC method has the advantage of being able to deal with a large amount of noise. These methods also have some disadvantages. These methods do not perform well with datasets that have complex geometries, and HT is sensitive to the selection of surface parameters.

### 2.3. Region Growing-Based Method

A lot of segmentation research has been undertaken based on the region growing method. Besl et al. used variable-order high-order polynomials as the surface fitting functions, and the point cloud was segmented by the seed point expansion method [48]. However, the segmentation of irregular complex surfaces needs to be improved. Rabbani et al. proposed a growth algorithm based on smooth constraints for segmenting point cloud data into smooth surfaces [49]. Vo et al. proposed an octree-based region growing method for point cloud segmentation with two stages based on a coarse-to-fine concept [41]. In general, the segmentation method based on regional growth can realize point cloud data segmentation, but the selection of seed points and parameters still requires human intervention and determination. The parameter settings have a great influence on segmentation results, which are therefore unstable.

### 2.4. Other Segmentation Methods

There are many other point cloud segmentation methods, for example, Delaunay triangulation [50], wavelet transform [51], three-dimensional grid method [39], line tracking algorithm [45], and so forth. Höfle et al. proposed a new GIS workflow with a decision tree and artificial neural network (ANN) classifier from LiDAR data for urban vegetation mapping [52]. Niemeyer et al. integrated a random forest classifier into a Conditional Random Field (CRF) framework, with which main buildings (larger than 50 m^2^) can be detected very reliably [53].

The application and research of laser scanning technology are not limited to the field of geoscience and mapping, and scholars who are engaged in computer and robot research also use laser scanning for robot environment perception and navigation research. These methods are mainly based on the classification of statistical learning supervision, which needs to learn the sample data in advance to determine the model parameters and then uses the resulting model to classify the unknown data. Anguelov et al. [54] and Triebel et al. [55] provided a valuable reference for automatic classification and filtering of ground point cloud data based on machine learning.

In general, in the field of laser point cloud data segmentation, scholars have carried out a lot of research. The main methods are clustering-based, model fitting-based, region growing-based methods, and others, and these methods have achieved certain research results. However, most of these segmentation methods are only applicable to a specific problem or data. Most parameters of the segmentation methods rely on manual experience, and the chosen parameters usually have a notable influence on the segmentation results. Meng et al. reviewed the LiDAR ground filtering algorithms and found that most filtering algorithms iteratively modify the neighborhood size to improve filtering accuracy in practice [56].

There are fewer methods that can be used for automatic segmentation. In this paper, an automatic parameter estimation method is proposed based on DBSCAN.

## 3. Methodology

**Figure 1:**
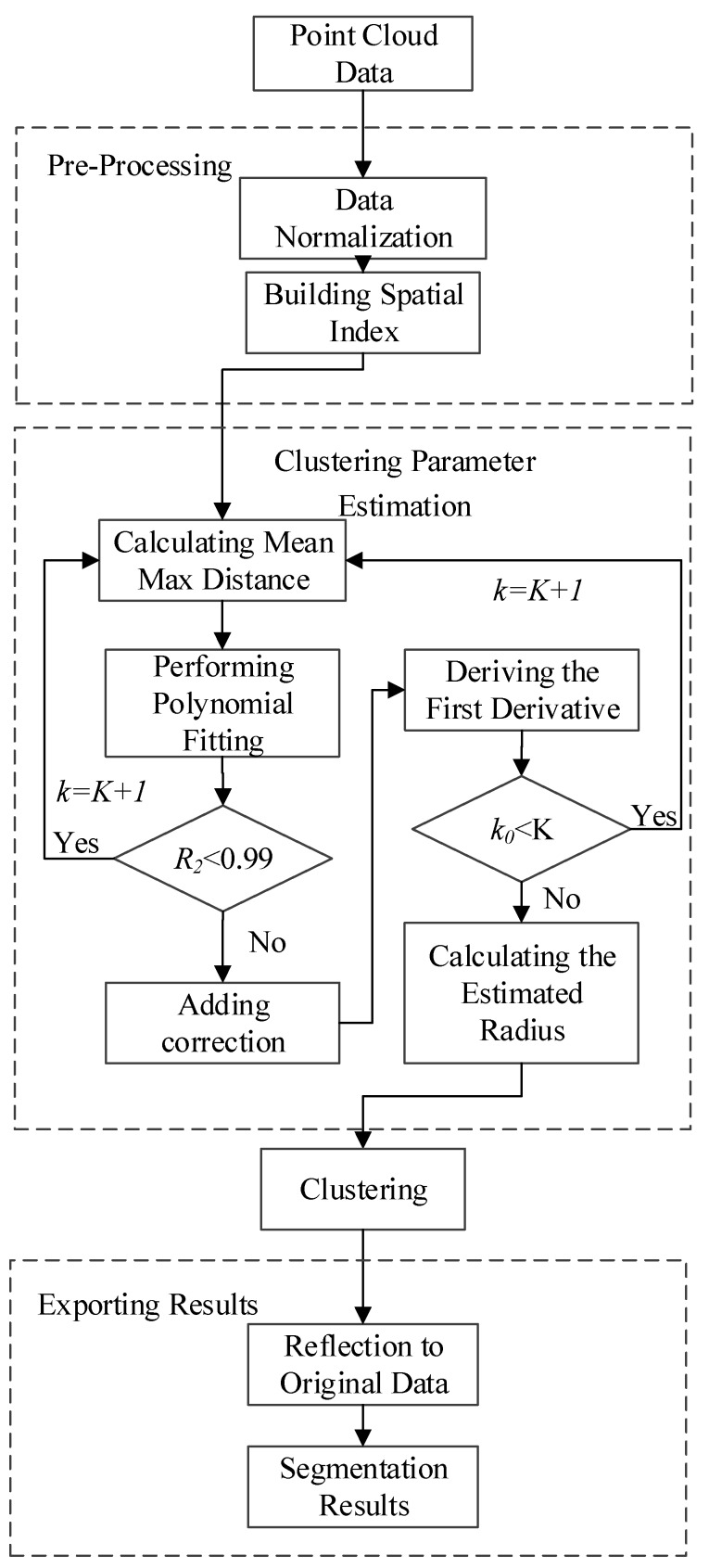
Segmentation workflow.

The estimation method based on average of k nearest neighbors’ maximum distance includes six steps: Data normalization, spatial index building, clustering parameter estimation, clustering, reflection to original data, and output results, as shown in Figure 1. The input data for the segmentation methodology are the data after registration, noise reduction, and coordinate transformation processing.

### 3.1. Pre-Processing

#### 3.1.1. Data Normalization

The point cloud data usually includes position (*X*, *Y*, *Z*) and intensity (*i*) data, and some may have color (*R*, *G*, *B*) data. These data have different units and dimensions. In order to make dimensions with different units suitable for comparison, it is necessary to perform data normalization before clustering. If only position data are considered for segmentation, data normalization is not necessary.

For the point cloud with n points each point has m dimensions, as shown in Equation (1):

(1) $$\left[\begin{array}{ccc} X_{11} & \cdots & X_{1m}\\ \vdots & \ddots & \vdots \\ X_{n1} & \cdots & X_{nm} \end{array}\right]$$

where n is the number of points in the cloud, and m is the number of dimensions. Then, the normalized value $Z_{ij}$ for the original value $X_{ij}$ is shown in Equation (2).

(2) $$Z_{ij}=\frac{x_{ij}-x_{j}}{\delta_{j}}\;i=1,2,\cdots ,n;\;j=1,2,\cdots ,m$$

where $\delta_{j}=\sqrt{\frac{1}{n-1}\overset{n}{\underset{i=1}{\sum }}{\left(x_{ij}-x_{j}\right)}^{2}}$ is the standard deviation of the sample, and $x_{j}=\frac{1}{n}\overset{n}{\underset{i=1}{\sum }}x_{ij}$ is the mean of the sample.

The normalized data is used for parameter estimation and cluster segmentation. Its relation to the original data is considered when the final results are generated.

#### 3.1.2. Definition of Distance in Clustering

In this study, the Euclidean distance is selected as the distance measure between the points. On the basis of the Euclidean distance, different variables can be set a given weight w according to their importance, as shown in Equation (3). For LiDAR point cloud data, different weights can be set for the spatial position, color information, and intensity. In this study, different weight settings are not used, and all weights are set to 1.

(3) $$d\left(p_{i},q_{j}\right)=\sqrt{w_{1}{\left(x_{i1}-x_{j1}\right)}^{2}+w_{2}{\left(x_{i2}-x_{j2}\right)}^{2}+\cdots +w_{m}{\left(x_{im}-x_{jm}\right)}^{2}}$$

where $p_{i}=\left(x_{i1},x_{i2},\cdots ,x_{im}\right)$ and $q_{j}=\left(x_{j1},x_{j2},\cdots ,x_{jm}\right)$ are two m dimension points in point cloud P, and $w_{m}=\left(w_{1},w_{2},\cdots ,w_{m}\right)$ is the given weight for each dimension.

In order to improve the computation efficiency, the squared distance between points is calculated in the actual distance calculation and comparison process.

#### 3.1.3. Kd-Tree Spatial Index

Spatial search is used frequently in the clustering process. An efficient indexing mechanism has to be established in order to speed up the search speed of massive points. In this paper, the Kd-tree [49] is used to establish the spatial index, which is an effective method for indexing multidimensional data. Point cloud data usually contain multi-dimensions (e.g., *x*, *y*, *z*, *r*, *g*, *b*, *intensity*). The value for *k* in the Kd-tree depends on the number of fields which are used for clustering. For example, the *k* value is 3 for a dataset with *x*, *y*, *z* fields and 6 for a dataset with 6 fields (*x*, *y*, *z*, *r*, *g*, *b*).

The Kd-tree index is mainly used in two operations in clustering: One is range search, and the other is K-Neighbor search. The range search is used to search the points which are inside a certain distance of a given point. The K-Neighbor search is to search the k points that are the nearest points to the given point.

### 3.2. Parameter Estimation

In the density-based clustering method, the degree of similarities between objects determines whether these objects belong to the same class or not. Hence, the selection of the criteria used for determination is of great importance to the clustering results.

The DBSCAN method is very sensitive to the input clustering threshold ε, and a small difference may lead to a completely different clustering result.

At present, the conventional way to set the clustering radius generally depends on human experience. Some researchers have focused on parameter estimation generally based on a certain kind of data, but for other data, the experience value may be not suitable. The open source software PCL (Point Cloud Library) for different data segmentations requires different parameters, and the recommendations are: Constantly try 5 times, 10 times, 15 times, 20 times and so forth for point cloud resolution until the best clustering results are found [57]. At the same time, the best parameters of different data are generally different, and the obtained parameters are difficult to reuse. Therefore, it is necessary to establish a clustering parameter estimation method for different point cloud data types.

In view of the above problems, the parameter estimation method based on the Average of K nearest neighbors’ maximum distance is proposed.

#### 3.2.1. Definition

Before introducing the method, two concepts must be defined.

**Point p’s KNN Max Distance** ($d_{maxi}$): For the point cloud data P with m points $p_{i}\left(i=1,2,3,\ldots \ldots ,m\right)$. Q is the collection of $p_{i}$’s nearest k points $q_{j}\left(j=1,2,3,\ldots \ldots ,k\right)$. $d\left(p_{i},q_{j}\right)$ is the distance between $p_{i}$ and $q_{j}$. Then, *p_i_*’s KNN max distance $d_{maxi}$ is defined as follows:

(4) $$d_{maxi}=\underset{1\;\leq \;j\;\leq \;k}{max}d_{ij}$$

**Figure 2:**
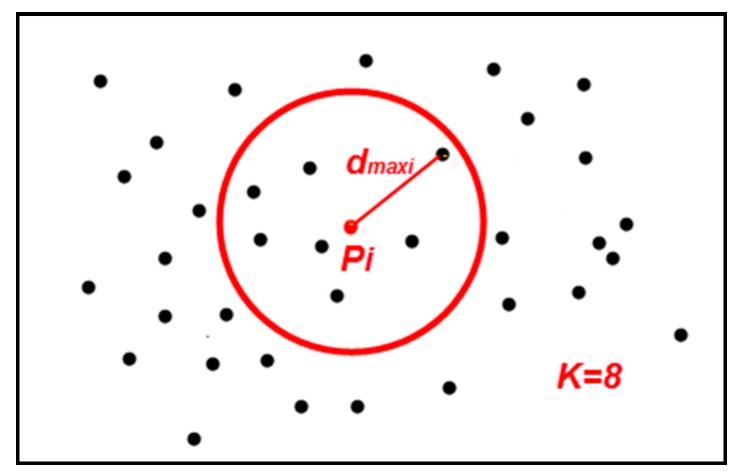
The point $p_{i}$’s KNN max distance $d_{maxi}\;\left(k=8\right)$.

In Figure 2, for the point $p_{i}$, when k=8, the 8 nearest points to $p_{i}$ are selected (including $p_{i}$ itself) by KNN search, and the distance between the farthest point and $p_{i}$ is $p_{i}$’s KNN max distance $d_{maxi}$.

**Point cloud P’s KNN mean max distance (**$D_{k}$**):** For the point cloud P with m points and given *k*, the point cloud P’s KNN mean max distance can be defined as follows:

(5) $$D_{k}=\sum_{i=1}^{m}d_{maxi}/m=\sum_{i=1}^{m}MAX\left(d_{ij}\right)/m$$

#### 3.2.2. Analysis

For an ideal scenario of a uniformly distributed point cloud, the relationship between $d_{maxi}$ and k may be similar to the circle area calculation formula:

(6) $$A=\pi R^{2}$$

where A is the area of a circle with radius R. For the uniformly distributed point cloud and the definition of $d_{maxi}$, k corresponds to A and $d_{maxi}$ corresponds to R. The relationship between k and $d_{maxi}$ can be described as follows:

(7) $$k=\pi \;d_{maxi}{}^{2}+f\left(k\right)$$

where f(k) is the correction from theoretical value to actual value.

Then:

(8) $$d_{maxi}={\left(\frac{k-f\left(k\right)}{\pi }\right)}^{\frac{1}{2}}$$

(9) $$D_{k}=\sum_{i=1}^{m}{\left(\frac{k-f\left(k\right)}{\pi }\right)}^{\frac{1}{2}}/m\;i=1,2,3,\ldots \ldots ,m$$

Therefore, based on the above analysis, the relationship between $D_{k}$ and k can be described by a polynomial fitting function.

**Figure 3:**
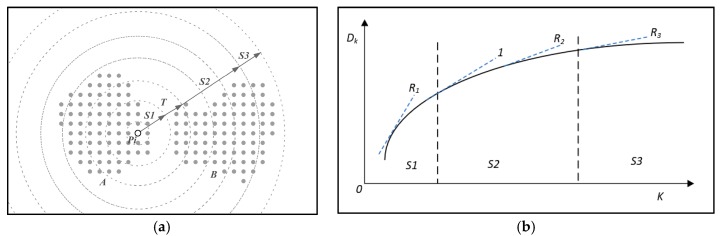
Analysis of the three stages of point $P_{i}$’s neighbor points. (**a**) Three stages and the turning point; (**b**) Tangent slopes for three stages and the turning point.

As k increases from 2→+∞, the fitting curve of $D_{k}$ and k have the following regular pattern, as shown in Figure 3a:

**Stage 1(S1)**: Point $p_{i}$’s neighbor points are mainly in one object.

The $D_{k}$ increases gradually with the increase of k with rate $R_{1}$.

**Stage 2(S2)**: Point $p_{i}$’s neighbor points are mainly in many nearby objects.

The $D_{k}$ increases with rate $R_{2}$ which is lower than $R_{1}$.

**Stage 3(S3)**: Point $p_{i}$’s neighbor points are the points in the whole dataset.

The limitation of $D_{k}$ may be a constant when the k→+∞ with rate $R_{3}$.

(10) $$\underset{k\to +\infty }{\lim}D_{k}=D_{max}$$

where $D_{max}$ is the distance between the two farthest points in the dataset.

Since the DBSCAN method segments points in the neighborhood to clusters, the optimal radius can be set to the value of $D_{k}$ when the stage changes from stage 1 to stage 2. The tangent slope of the curve can be used as a way to find the turning point (*T* in Figure 3a) from stage 1 to stage 2. Corrections can be added to the fitting curve to make it so that $D_{k}$ and k have the same range. After adding corrections, the tangent slopes for each stage are $R_{1}>1,\;R_{2}<1,R_{3}<1$, as shown in Figure 3b. Therefore, the turning point from stage 1 to stage 2 can be found when the tangent slope R=1.

In the fitting curve, a different first derivative value corresponds with a different distance value. When the first derivative is set equal to 1, the corresponding $D_{k}$ is the optimal value for radius ε.

#### 3.2.3. Method

**Figure 4:**
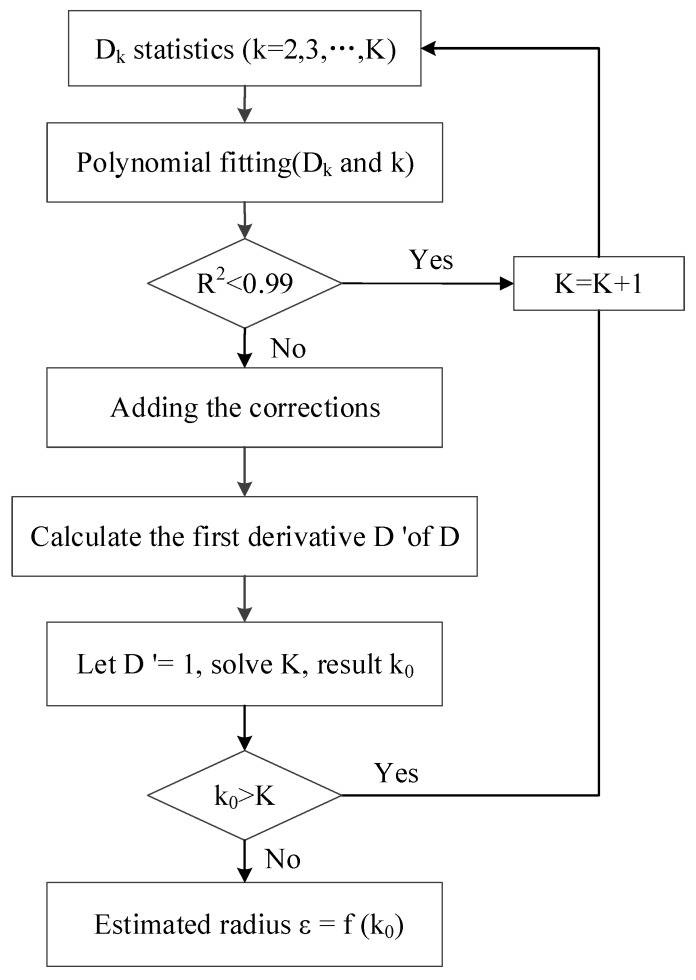
Parameter estimation process.

The detailed process of the method is shown in Figure 4:

(1) Calculating Point Cloud P’s KNN mean max distance ($D_{k}$)

When k=1, the nearest point of the point P is the point P itself, the distance is 0, so the value of k is k∈[2,K]. Calculate $d_{k}$ according to Equation (5) to obtain the discrete function of $d_{k}$; that is, the $\left(2,d_{m2}\right),\left(3,d_{m3}\right),\left(4,d_{m4}\right),\ldots ,\left(K,d_{mK}\right)$ sequence.

(11) $$d_{k}=g\left(k\right)$$

(2) Performing the polynomial fitting for the discrete function $d_{k}$

The polynomial fitting for Equation (12) is performed to obtain the continuous function $D_{k}$:

(12) $$D_{k}=f\left(k\right)\;k\in \left[2,K\right]$$

If $R^{2}<0.99$ then K=K+1, and repeat Step 1.

(3) Adding corrections

Let K be the maximum value of k, and $D_{kmax}$ be the maximum value of the $D_{k}$, then add the correction number $\frac{K}{D_{kmax}}$

(13) $$D_{mk}=\frac{K}{D_{kmax}}\cdot f\left(k\right)k\in \left[2,K\right]$$

(4) Deriving the first derivative of $D_{mk}$:
  (14) $$D_{mk^{\prime }}=\frac{K}{D_{kmax}}\cdot f^{\prime }\left(K\right)$$

Let $D_{mk^{\prime }}$ = 1, solve $k=k_{0}$. If $k_{0}>K$ then K=K+1 and repeat steps 1 to 4.

(5) Calculating the estimated radius ε

Substitute $k=k_{0}$ into Equation (13) to get $D_{k}=f\left(a\right)$, then $\varepsilon =D_{k}$ is the estimated radius.

The distances between points in the point cloud are analyzed and the relationship between k and f(k) is derived. When the tangent slope of the function is set to 1, the corresponding value f(k) of k is considered as the optimal clustering radius. The effectiveness and accuracy of the method are verified through experiments in Section 4.

### 3.3. Cluster Segmentation

DBSCAN is a density-based clustering algorithm that does not require the specification of the cluster number in the data, unlike k-means. DBSCAN can find arbitrarily shaped clusters, and this characteristic makes DBSCAN very suitable for LiDAR point cloud data. The DBSCAN algorithm is used for point cloud segmentation in this study.

#### 3.3.1. Parameters

Especially for high-dimensional data, the so-called “curse of dimensionality” makes it difficult to find an appropriate value for threshold ε. This effect, however, also exists in other algorithms based on Euclidean distance [14]. In this study, the improved DBSCAN algorithm can deal with high-dimensional data well, including normalized high-dimensional data and the Kd-tree index.

DBSCAN requires just two parameters: *minPts* and ε. In this study, another parameter, *maxPts,* is added to control the size of clusters. *MinPts* and *maxPts* are selected according to the point number that the smallest and biggest objects may have. The value of *minPts* will affect the small objects to be clusters or noises; the *maxPts* will affect how big the objects may be before being considered as one cluster instead of being split apart. These two parameters have to be set manually in this study. Parameter ε can be calculated by the method proposed above.

#### 3.3.2. Clustering

In HDBSCAN (Hierarchical DBSCAN) [58] the concept of border points was abandoned, and only core points are considered to be part of a cluster at any time, which is more consistent with the concept of a density level set. Rusu also proposed an improved clustering method based on DBSCAN that uses only core points [57]. In this study, the DBSCAN algorithm is improved as follows (Algorithm 1):

**Algorithm 1** Improved DBSCAN AlgorithmInput: Dataset: *P*, *minPts*, ε, *maxPts*Output: Clusters *C***1** Setting up an empty list of clusters *C* and an empty queue *Q* for the points that need to be checked **2 for all**$p_{i}\in P$, do**3  if**$p_{i}$ is processed **then****4**    continue**5**  **end****6**   add $p_{i}$ to the current queue *Q***7**   **for all**$p_{j}\in Q$ do**8**     search for the set $p_{j}^{k}$ of point neighbors of $p_{j}$ in a sphere with radius r<ε;
**9     for all**
$p_{t}\in p_{j}^{k}$
**10       if**$p_{t}$ is not processed **then****11**         add $p_{t}$ to *Q*
**12       end**

**13     end**

**14   end**
**15**   **n** = the point number of *Q***16**   **if**
n>minPts and n<maxPts
**then****17**     add *Q* to the list of clusters *C*
**18**     **for all**
$p_{j}\in Q$
**do****19**       mark $p_{j}$ processed**20**     **end**21     reset *Q* to an empty list**22**   **end**
**23 end**

**24 Return**
*C*


### 3.4. Exporting Segmentation Results

It’s necessary to reflect the normalized data to the original data for the output result because all the processes are undertaken on the normalized data. The point number and sequence are kept unchanged in both the normalized data and original data, so it is possible to get the original data and export the segmentation result to data files of certain formats.

## 4. Experimental Results and Analysis

In order to test the robustness and accuracy of the method, experiments on airborne and mobile LiDAR data were performed with both spatial information and the combination of spatial information and color information.

### 4.1. Airborne LiDAR Data Experiments

#### 4.1.1. Study Area and Data Source

The study area of the airborne LiDAR data is located in the city of Baltimore, Maryland, USA, and the data were downloaded from the NOAA Coastal Services Centre (https://coast.noaa.gov/htdata/lidar1_z/). The data were acquired by a Leica Airborne Laser Scanner Model ALS 50, which was used in a Sanborn Aero Commander 500B to acquire the data. The flying height was 1400 m, the scan frequency was 36 KHz, the pulse rate was 63 KHz, and the point density was 1.0 m. The original point cloud data does not have color information, therefore data fusion with remote sensing images was performed to add this color information.

**Figure 5:**
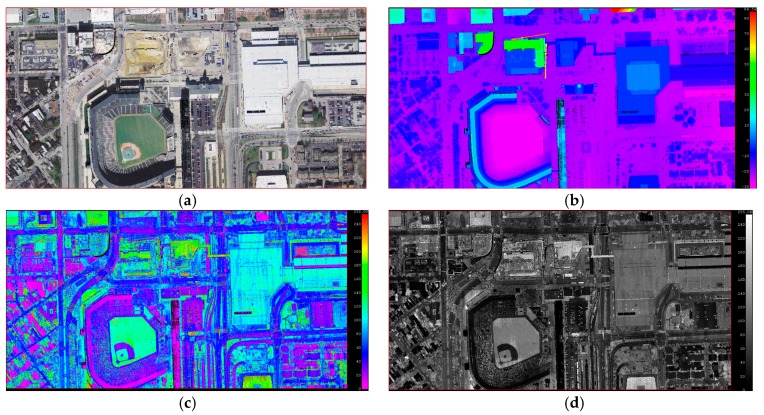
Original data. (**a**) Colored by RGB; (**b**) colored by height; (**c**) colored by intensity; (**d**) grayed by intensity.

**Figure 6:**
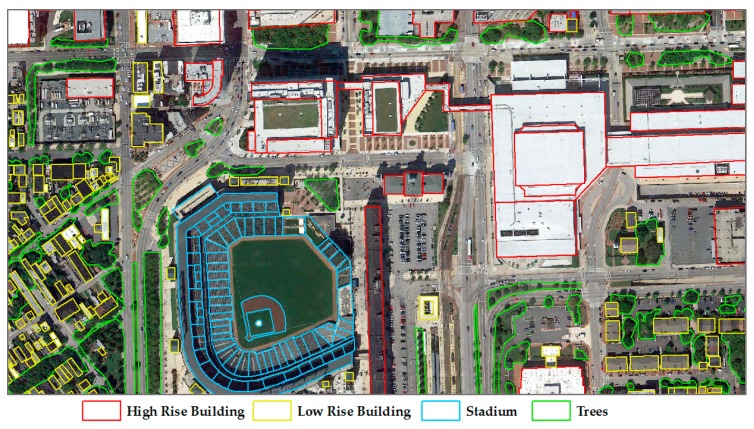
Remote sensing image and reference data.

The study area includes sports grounds, roads, high-rise buildings, low-rise buildings, trees, and so forth. The point cloud data have a spatial position, echo intensity, and color information. The original point cloud data is shown in Figure 5, and the corresponding remote sensing image data and reference data are shown in Figure 6. The reference data were collected by the authors based on the remote sensing images.

Although the DBSCAN algorithm can deal with noisy data, we still had the data filtered in order to achieve a more accurate statistical result. After noise removal, the point number of the point cloud is 3,388,214.

It is necessary to combine the reflection intensity information with spatial location information, color information, and so forth to improve the segmentation accuracy. In this study, after the analyses of the data, the reflective intensity of trees and buildings are closer compared to the spatial and color information in the experiment data. Therefore, if the reflective intensity information is involved in clustering segmentation, the distance between classes—such as trees and buildings—will be reduced, which will affect the segmentation accuracy. For this reason, spatial position and color information are chosen to participate in point cloud data segmentation.

In order to evaluate the accuracy of segmentation, reference data is collected from the remote sensing images. High rise buildings, low rise buildings, stadiums, and trees are collected for the reference data, as shown in Figure 6. There are 333 reference objects collected.

#### 4.1.2. Using Spatial Information

##### Parameter Estimation

**Figure 7:**
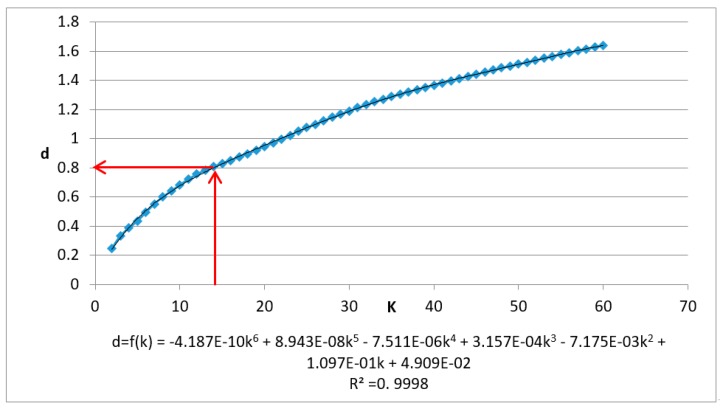
Chart and fitting curve of the mean max distance of *k* nearest neighbor of airborne laser scanning data using *X*, *Y*, *Z* fields.

The test data are first normalized and the Kd-tree spatial index is built. When K=60, $R^{2}>0.99$ and $k_{0}<K$, and the data’s KNN mean max distance ($D_{k}$) is calculated when *k* = (2,3,4,…,60). The results and fitting polynomial are shown in Figure 7. The detailed process is as follows.

Adding corrections, when K=60, $D_{k}=1.64151$, the polynomial fitting curve is shown as follows:

(15) $$D_{mk}=\frac{60}{1.64151}\times f\left(k\right)$$

Let the first derivative:

(16) $$D\_mk^{\prime }=60/1.64151\times f^{\prime }\;\left(k\right)=1$$

Solve $k_{0}$ = 14.389, then the estimated parameter $r_{0}=f\left(k_{0}\right)=0.8114$.

(The estimated value of the threshold ε and the corresponding k value has been marked with red lines. The fitting curve and variance are at the bottom of the graph.)

##### Clustering and Results

Different radii are selected for the clustering segmentation ε ∈(0.6,0.7,0.8, 0.8114, 0.9, 1.0,1.1) and all *minPts* = 100, *maxPts* = 3,000,000. The input parameters and the results (run time, number of clusters, and noise ratio) are shown in Table 1.

The resulting clusters are the clusters with a higher point count than *minPts*. The noise ratio is the noise proportion of the dataset total point number.

As can be seen from Table 1, the clustering time is gradually increasing with the increase of the cluster radius. The total number of clustering results is decreasing, and there is a downward trend in noise ratio. Most of the clustering results contain 200–4000 points. When the estimated parameter ε = 0.8114, the clustering results are distributed in the range of 200–50,000, and the noise ratio is 3.9%.

**Figure 8:**
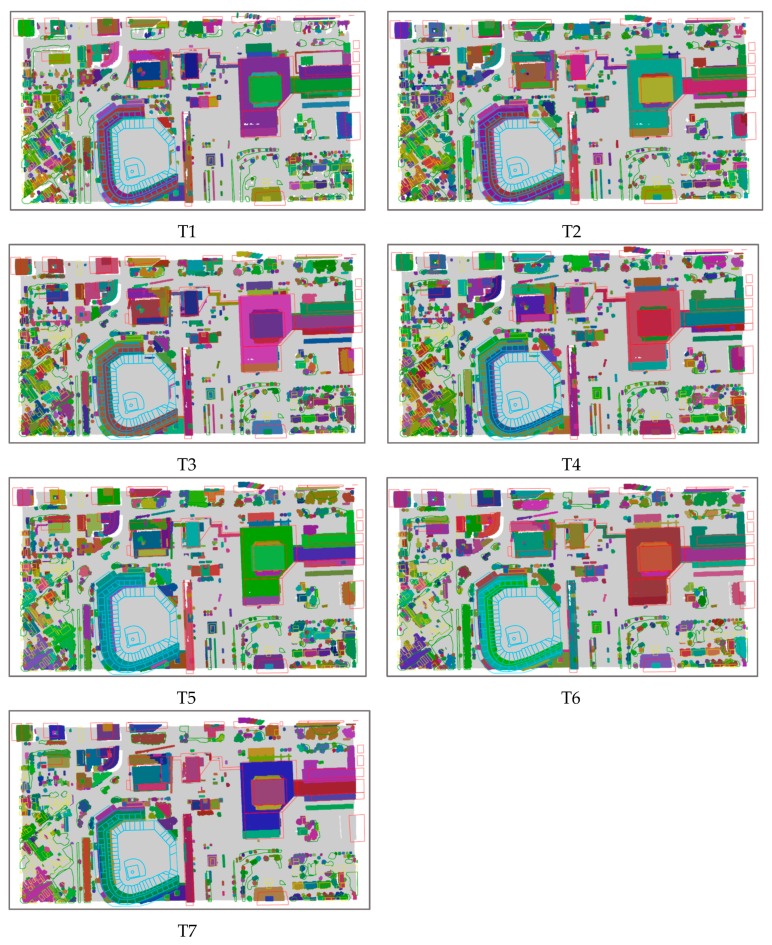
Airborne laser scanning data segmentation result maps using *X*, *Y*, *Z* fields.

The experimental results are shown in Figure 8.

It can be seen that the results change from the fragmented state to the merged state with the increase of the radius of the cluster. If the radius is less than the estimated value, as in Test T1, T2, and T3, the segmentation results are fragmented. The reason for this is that many objects are over-segmented. For example, the buildings to the west of the baseball field are segmented into many blocks. When the radius is greater than the estimated value, many different objects are segmented well. For example, in Test T6 (ε = 1.0) and T7 (ε = 1.1), low-rise buildings in the lower left corner of the road and vegetation are segmented into one cluster. In Test T4 (ε = 0.8114), high-rise buildings, low-rise buildings, and some vegetation have been clearly segmented. Compared to the other segmentation results, although there are some objects that are still over-segmented or under-segmented, it is a satisfactory result.

##### Accuracy Evaluation

Hoover et al. divided point cloud segmentation results into five categories according to the segmentation effect: Correct detection, over-segmentation, under-segmentation, missed, and noise [59]. This criterion is used for accuracy evaluation in this study.

Over-segmentation means one object is segmented into multi-parts, while under-segmentation means the segmentation is insufficient—objects nearby are segmented into one. Missed means objects are missed in the segmentation results. The goal of point cloud data segmentation is to minimize the occurrence of the last four error divisions.

**Figure 9:**
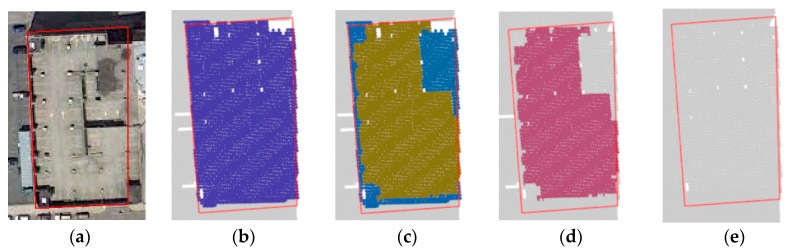
Evaluation criteria. (**a**) Reference building image; (**b**) correct detection; (**c**) over-segmentation; (**d**) under-segmentation; (**e**) missed.

Figure 9 shows a reference building and the four segmentation results in different tests, except noise. If the number of points within a cluster is less than *minPts*, all the points in the cluster are considered to be noise in the tests.

In this study, we focus on the segmentation of different classes. Therefore, in the accuracy evaluation of the segmentation results, the same class of objects segmented into one cluster is considered a correct detection and not under-segmentation. Under-segmentation is a cluster with objects of different classes. For example, a cluster with several trees is correct detection, but one with trees and buildings is under-segmentation.

Each test result is evaluated according to the referenced data and the accuracy is shown in Table 2. The accuracy of Test T4, which used the estimated parameter of 75%—higher than the parameters that were estimated. In Test T1, many objects are considered as noise or over-segmented, and that leads to low accuracy. In Test T7, missed objects are the main factor for low accuracy.

#### 4.1.3. Using Spatial and Color Information

##### Parameter Estimation

**Figure 10:**
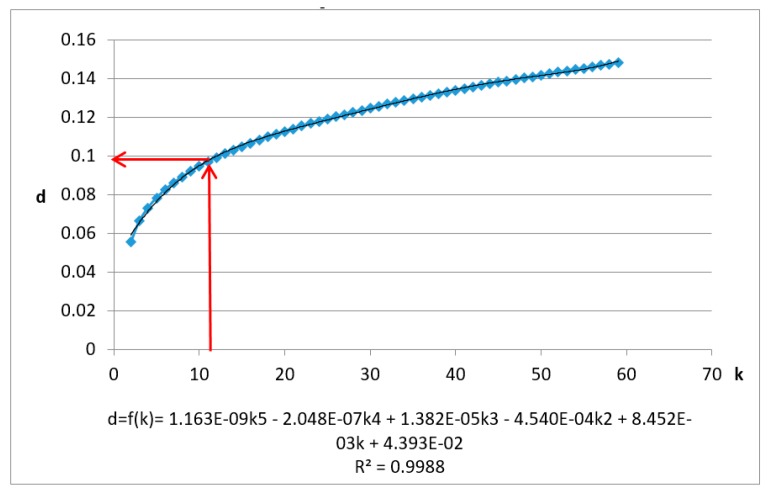
Chart and fitting curve of the mean max distance of k nearest neighbor of airborne laser scanning data using *X*, *Y*, *Z*, *R*, *G*, *B* fields.

The LiDAR data with spatial and color information, including six dimensions, were normalized and the Kd-tree spatial index was built. When K=60, $R^{2}>0.99$ and $k_{0}<K$. The data’s KNN mean max distance ($D_{k}$) is calculated when k=(2,3,4,⋯,60). The results and fitting polynomial are shown in Figure 10. The detailed process is as follows:

Adding corrections, if K=60, $D_{k}=0.148$, the polynomial fitting curve is shown as follows:

(17) $$D_{mk}=\frac{60}{0.148}\times f\left(k\right)$$

Let the first derivative:

(18) $$D\_mk^{\prime }=60/0.148\times f^{\prime }\;\left(k\right)=1$$

Solve $k_{0}$ *=* 10.860, then the estimated parameter $r_{0}=f\left(k_{0}\right)=0.097$.

(The estimated value of the threshold ε and the corresponding k value have been marked with red lines. The fitting curve and variance are at the bottom of the graph).

##### Clustering and Results

Different radii are selected for the clustering segmentation ε∈(0.07,0.08,0.09, 0.097, 0.10, 0.11,0.12), and all *minPts* = 100, *maxPts* = 3,000,000. The input parameters and the results (run time, number of clusters and noise ratio) are shown in Table 3.

As can be seen from Table 3, the clustering time is gradually increasing with the increase of the cluster radius. The total number of clustering results is decreasing, and there is a downward trend in noise ratio.

**Figure 11:**
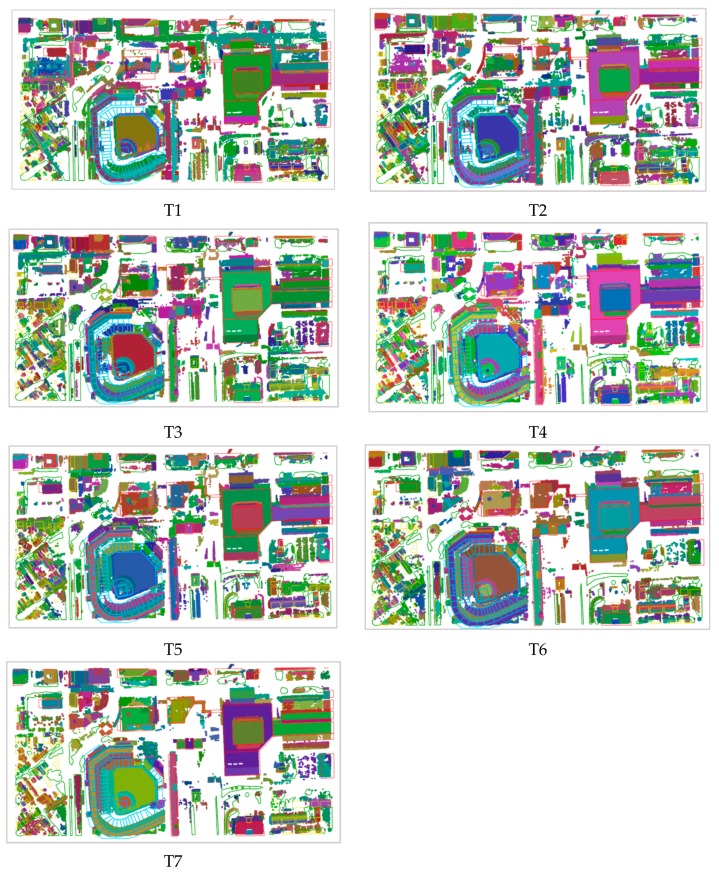
Airborne laser scanning data segmentation result maps using *X*, *Y*, *Z*, *R*, *G*, *B* fields.

Most of the clustering results contain 200–2000 points. If the estimated parameter ε = 0.097, the clustering results are distributed in the range of 200–50,000 points and the noise ratio is 14.4%. The results are shown in Figure 11.

When ε < 0.097, for example in Test T1 (ε = 0.07), the main high-rise buildings have been separated; the sports field grass is divided, the road is divided into six categories, the top of the stadium is divided into three categories, and some low-rise buildings and vegetation grassland are divided together into one class. Consequently, if ε < 0.097, some objects are over-segmented, while others are under-segmented.

In Test T4 (ε = 0.097), the high-rise building roof and part of the low-rise buildings mixed with trees have been separated, the roads and the green belt in the middle of roads are also separated, and the grass field, the runway, and different areas of the seats are also separated. It can be seen that when ε = 0.09, ε = 0.097 or ε = 0.10, there are less over or under-segmentation cases, and the segmentation results are better than in T1, T2, and T3.

When ε > 0.097, for example in Test T7 (ε = 0.12), the main roads and trails are not separated, and low-rise buildings, the grass field in the sports ground and the runway have not been separated. In general, in Tests T5, T6, and T7, most objects are under-segmented.

##### Accuracy Evaluation

Each test result is evaluated according to the referenced data and the accuracy is shown in Table 4. There are 333 objects in the referenced data. The accuracy rate of Test T4 that uses the estimated value of ε is highest at 74%.

### 4.2. Mobile LiDAR Data Experiments

#### 4.2.1. Study Area and Data Source

**Figure 12:**
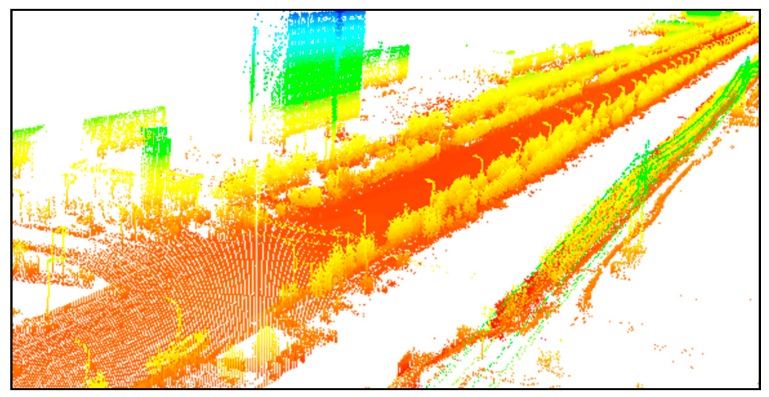
Original mobile point cloud data.

The study area is a 500 m long street with trees, street lamps, buildings, and other objects, as shown in Figure 12. The data were acquired by the Optech Lynx V100 mobile survey system. The sampling frequency was 75 Hz and the laser measurement rate was 100 kHz. Vehicle speed along this road was 40 km/h. The point spacing was 2 to 3 cm at 10 m.

**Figure 13:**
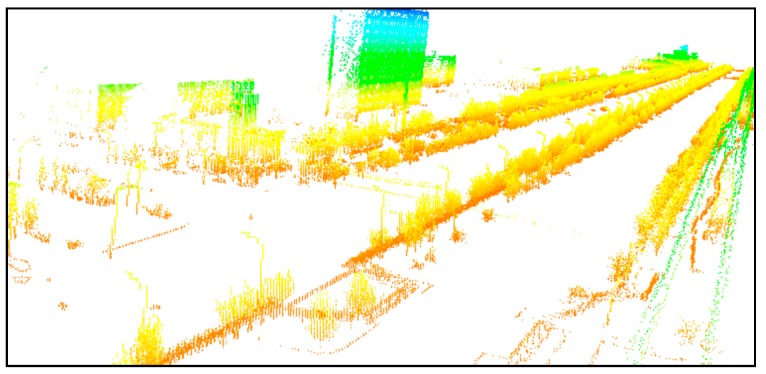
Point cloud without ground points.

The data have both spatial and intensity information. The number of points is seven million, and most of the points on the road surface are more intensive than those on trees, buildings, street lamps, and so forth. The intensive points are very important to the road surface quality inspection, but for the purpose of ground object segmentation, the ground points have to be removed in order to reduce the influence of different densities in the clusters. The rest of the data containing trees, street lamps, and buildings were used for segmentation. A horizontal plane based off the lowest points, and then a buffer above the plane, was used to classify the points within the buffer as ground. We developed a C# tool to read the PCD (Point Cloud Data) file and remove the ground points. After ground point removal, the remaining point number was 854,994, as shown in Figure 13.

For one class of objects, specifically trees, and light lamps, the reflective intensity information and color information have almost the same value. If they are involved in clustering segmentation, the distance between objects will be reduced, which will affect the segmentation effect. Therefore, for the mobile LiDAR data, a spatial position was chosen to participate in the point cloud data segmentation.

**Figure 14:**
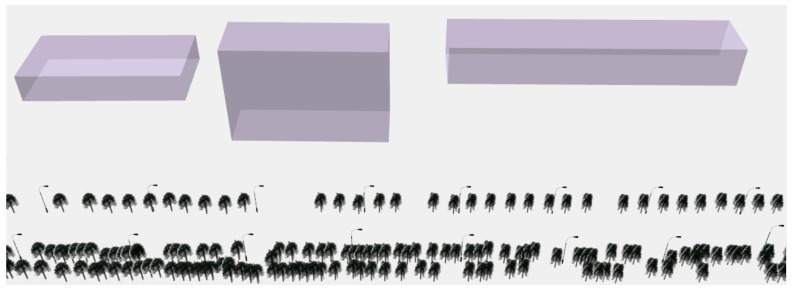
Part of the reference data.

The reference data were collected by the authors for accuracy evaluation based on the LiDAR data using ESRI ArcScene 10.3. The reference data contain trees, street lamps, and buildings, and the numbers are 807, 94, and 18, respectively. Part of the reference data are shown in Figure 14.

#### 4.2.2. Using Spatial Information

##### Parameter Estimation

**Figure 15:**
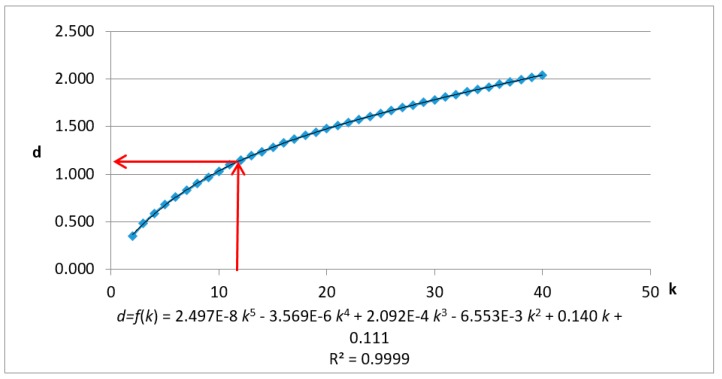
Chart and fitting curve of the mean max distance of the k nearest neighbor of mobile laser scanning data using *X*, *Y*, *Z* fields.

The data with spatial information were normalized and the Kd-tree spatial index was built with three dimensions. When K=40, $R^{2}>0.99$ and $k_{0}<K$. The data’s KNN mean max distance ($D_{k}$) is calculated when k=(2,3,4,⋯,40). The results and fitting polynomial are shown in Figure 15. The detailed process is as follows:

Adding corrections, when K=40, $D_{k}=2.04$, the polynomial fitting curve is shown as follows:

(19) $$D_{mk}=\frac{40}{2.04}\times f\left(k\right)$$

Let the first derivative:

(20) $$D\_mk^{\prime }=40/2.04\times f^{\prime }\;\left(k\right)=1$$

Solve $k_{0}$ *=* 12.063, then the estimated parameter $r_{0}=f\left(k_{0}\right)=1.14686$.

(The estimated value of the threshold ε and the corresponding *k* value has been marked with red lines. The fitting curve and variance are at the bottom of the graph).

###### Clustering and Results

Different radii were selected for the clustering segmentation ε∈(0.5,0.8,1.1,1.14686,1.2,1.5,1.7). All *minPtss* were set to 200 and *maxPtss* to 854,994. The input parameters and results (run time, number of clusters, and noise ratio) are shown in Table 5.

As can be seen from the table, the clustering time gradually increases as the cluster radius increases. The total number of clustering results is decreasing, and correspondingly the noise ratio has a downward trend. Most of the clustering results contain 100–3000 points. In Test T4 (ε = 1.14686), the clustering results are distributed in the range of 100–60,000 points, and the noise ratio is 14.4%.

**Figure 16:**
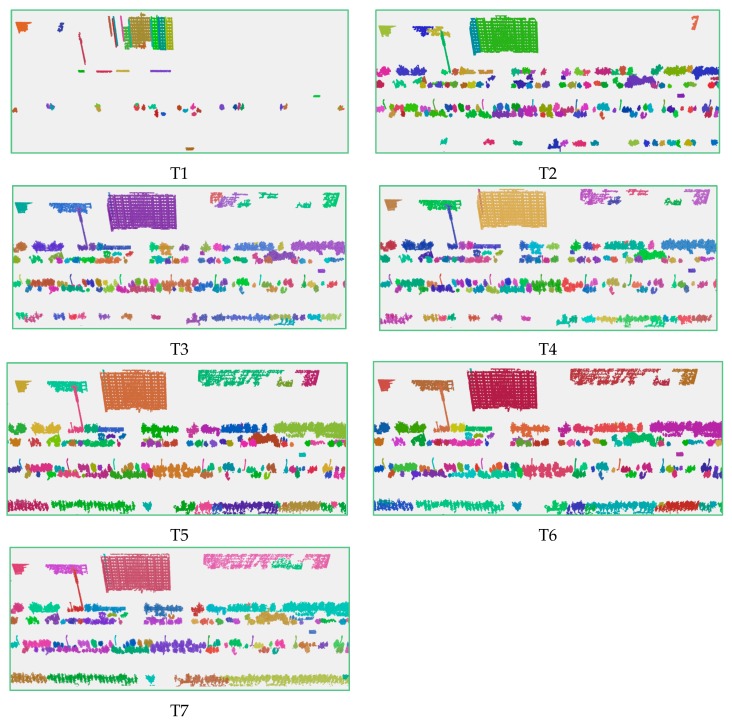
Part segmentation results of the mobile laser scanning data using *X, Y, Z* fields.

The experimental results are shown in Figure 16.

It can be seen from the results graph that in Test T4(ε = 1.14686), most of the buildings, single trees, street lamps, etc., have been divided while some single trees in the row of trees have not been separated. It is because these trees are too close to each other to be segmented. With the increasing of the cluster radius, more street lamps and trees are segmented to one cluster because of under-segmentation. Such as Test T7 (ε = 1.7), only a few single trees have been segmented, most single trees are segmented into a row of trees, at the same time, more street lamps are segmented with trees, as shown in Figure 16, T6, and T7. When the radius is less than the estimated value, such as Test T2 (ε = 0.8), there are only a few single trees or rows of trees, less or no street lamps are segmented. This can be considered as over-segmented.

##### Accuracy Evaluation

The test results were evaluated against the reference data according to the evaluation standard in Section Accuracy Evaluation. If several trees are segmented to one cluster, the cluster is considered to be correct detection.

Each test result was evaluated according to the referenced data, and the accuracy is shown in Table 6. The accuracy rate of Test T4 that uses the estimated value of ε is 71%. It is higher than those of the tests that uses a value greater or less then the value estimated.

### 4.3. Results

**Figure 17:**
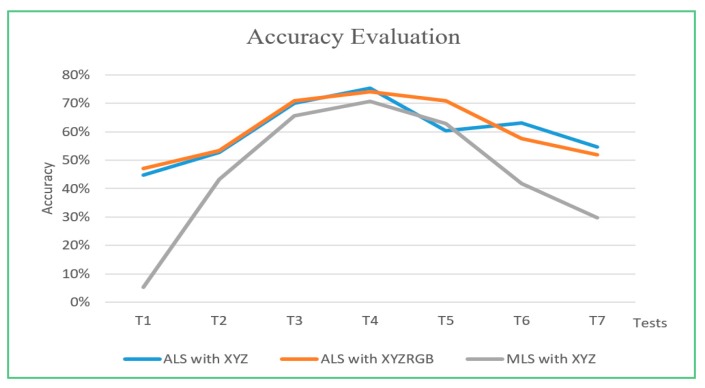
Accuracy graph for estimated ε (T4), radii gradually lower than ε (T3, T2, T1) and gradually greater than ε (T5, T6, T7).

Airborne LiDAR (ALS) and mobile LiDAR (MLS) data with spatial and color information are segmented using the estimated ε and parameters greater and less than ε. The accuracy of each segmentation test is evaluated according to the reference data. The results are shown in Figure 17.

The experimental results show that the point cloud can be segmented automatically by the proposed method based on spatial position and color feature. The accuracy rate using ε estimated by the proposed method is 75%, 74%, and 71%, which is higher than the accuracy using parameters greater or less than the estimated one used in this study.

In the ALS datasets, objects include the runway, lawn, high-rise, and low-rise building, roads, trees, and playground; in the MLS datasets, single trees, street lamps, and buildings are clearly segmented. The parameter estimation method can be used for automatic segmentation with higher accuracy.

## 5. Conclusions

A segmentation algorithm based on DBSCAN density clustering technology is proposed with a novel automatic parameter estimation method for the parameter ε, which is the critical parameter for the clustering process. The optimal clustering parameter ε can be calculated automatically according to the characteristics of the data, and the user need not have a good understanding of the data. This method uses the intrinsic properties of the point cloud data, analyzes the distance between points in the point cloud, and derives the relationship between k and the mean max distance f(k). When the tangent slope of the function is equal to 1, the corresponding f(k) value of k is considered as the optimal clustering radius.

The method was evaluated on different types of point cloud data, namely airborne and mobile data with and without color information. The experimental results show that the segmentation accuracy, using parameter ε values, estimated by the proposed method are 75%, 74%, and 71%, which are higher than those using parameters greater or less than the estimated one in this method.

The experimental results demonstrate the robustness of the parameter estimation method, which can also be applied to high-dimensional data. This method can be applied to airborne and mobile point cloud data processing systems, reducing manual workload, and improving the automation of data processing. This method changes the present situation, in which the setting of clustering parameters mainly depends on empirical values, and the data have to be well understood.

Future research could be focused on the estimation of another two parameters, *minPts* and *maxPts*, the beginning and ending condition of iteration segmentation. The expression and comparison of dispersed points and automatic object identification could be further researched based on the segmentation method proposed in this paper.

## Figures and Tables

**Table 1:** The segmentation results using different ε for airborne laser scanning data using *X*, *Y*, *Z* fields.

Class	Name	T1	T2	T3	T4	T5	T6	T7
Inputs	ε	0.6	0.7	0.8	0.8114	0.9	1.0	1.1
Results	Run Time (s)	20.0	22.2	22.2	22.0	22.8	26.5	32.8
	No. of Clusters	901	828	729	728	660	577	486
	Noise Ratio (%)	8.8	5.8	4.1	3.9	3.0	2.3	1.7

**Table 2:** Accuracy evaluation using different ε for airborne laser scanning data using *X*, *Y*, *Z* fields.

Test	ε	Correct Detection	Under-Segmentation	Over-Segmentation	Missed	Accuracy
T1	0.6	149	40	81	63	45%
T2	0.7	176	36	65	56	53%
T3	0.8	233	17	36	47	70%
T4	0.8114	251	21	15	46	75%
T5	0.9	201	33	36	63	60%
T6	1.0	210	29	26	68	63%
T7	1.1	182	51	7	93	55%

**Table 3:** The segmentation results using different thresholds for airborne laser scanning data using *X*, *Y*, *Z*, *R*, *G*, *B* fields.

Class	Name	T1	T2	T3	T4	T5	T6	T7
Inputs	ε	0.07	0.08	0.09	0.097	0.1	0.11	0.12
Results	Run Time (s)	52.1	65.2	82.0	91.6	105.4	133.3	166.3
	No. of Clusters	635	632	570	504	498	412	340
	Noise Ratio (%)	31.0	23.6	18.0	14.4	13.2	9.3	6.1

**Table 4:** Accuracy evaluation using different ε values for airborne laser scanning data using *X*, *Y*, *Z*, *R*, *G*, *B* fields.

Test	ε	Correct Detection	Under-Segmentation	Over-Segmentation	Missed	Accuracy
T1	0.07	157	53	73	50	47%
T2	0.08	178	58	58	39	53%
T3	0.09	236	27	48	22	71%
T4	0.097	247	24	43	19	74%
T5	0.10	236	25	40	32	71%
T6	0.11	192	65	25	51	58%
T7	0.12	173	90	8	62	52%

**Table 5:** The segmentation results using different thresholds for mobile laser scanning data from *X*, *Y*, *Z* fields.

Class	Name	T1	T2	T3	T4	T5	T6	T7
Inputs	ε	0.5	0.8	1.1	1.14686	1.2	1.5	1.7
Results	Run Time (s)	3.5	6.3	9.4	12.8	13.7	14.8	18.5
	Number of clusters	186	663	619	611	592	459	385
	Noise Ratio (%)	84.1	27.9	15.9	14.4	12.7	8.5	6.5

**Table 6:** Accuracy evaluation using different ε values of mobile laser scanning data using *X*, *Y*, *Z* fields.

Test	ε	Correct Detection	Under-Segmentation	Over-Segmentation	Missed	Accuracy
T1	0.5	48	14	4	846	5%
T2	0.8	394	93	3	422	43%
T3	1.1	598	51	23	240	66%
T4	1.14686	645	44	15	208	71%
T5	1.2	579	131	3	199	63%
T6	1.5	382	338	4	188	42%
T7	1.7	271	455	3	183	30%
